# Diversity-oriented natural product platform identifies plant constituents targeting *Plasmodium falciparum*

**DOI:** 10.1186/s12936-016-1313-7

**Published:** 2016-05-10

**Authors:** Jin Zhang, John J. Bowling, David Smithson, Julie Clark, Melissa R. Jacob, Shabana I. Khan, Babu L. Tekwani, Michele Connelly, Vladimir Samoylenko, Mohamed A. Ibrahim, Mohamed A. Zaki, Mei Wang, John P. Hester, Ying Tu, Cynthia Jeffries, Nathaniel Twarog, Anang A. Shelat, Larry A. Walker, Ilias Muhammad, R. Kiplin Guy

**Affiliations:** National Center for Natural Products Research, Research Institute of Pharmaceutical Sciences, School of Pharmacy, The University of Mississippi, University, MS 38677 USA; Department of Chemical Biology and Therapeutics, St. Jude Children’s Research Hospital, Memphis, TN 38105 USA; Department of Biomolecular Sciences and Research Institute of Pharmaceutical Sciences, School of Pharmacy, The University of Mississippi, University, MS 38677 USA; Genentech, San Francisco, CA USA; Keiser University, West Palm Beach, FL USA; Beni-Suef University, Beni-Suef, Egypt

**Keywords:** Drug discovery, Spectrometry, Natural products, Alkaloids, Terpenes, Antimalarial, *Plasmodiumfalciparum*, UPLC-ELSD-PDA-ESI–MS, *Berberis thunbergii*, *Eugenia rigida*

## Abstract

**Background:**

A diverse library of pre-fractionated plant extracts, generated by an automated high-throughput system, was tested using an in vitro anti-malarial screening platform to identify known or new natural products for lead development. The platform identifies hits on the basis of in vitro growth inhibition of *Plasmodium falciparum* and counter-screens for cytotoxicity to human foreskin fibroblast or embryonic kidney cell lines. The physical library was supplemented by early-stage collection of analytical data for each fraction to aid rapid identification of the active components within each screening hit.

**Results:**

A total of 16,177 fractions from 1300 plants were screened, identifying several *P. falciparum* inhibitory fractions from 35 plants. Although individual fractions were screened for bioactivity to ensure adequate signal in the analytical characterizations, fractions containing less than 2.0 mg of dry weight were combined to produce combined fractions (COMBIs). Fractions of active COMBIs had EC_50_ values of 0.21–50.28 and 0.08–20.04 µg/mL against chloroquine-sensitive and -resistant strains, respectively. In *Berberis thunbergii*, eight known alkaloids were dereplicated quickly from its COMBIs, but berberine was the most-active constituent against *P. falciparum*. The triterpenoids *α*-betulinic acid and *β*-betulinic acid of *Eugenia rigida* were also isolated as hits. Validation of the anti-malarial discovery platform was confirmed by these scaled isolations from *B. thunbergii* and *E. rigida*.

**Conclusions:**

These results demonstrate the value of curating and exploring a library of natural products for small molecule drug discovery. Attention given to the diversity of plant species represented in the library, focus on practical analytical data collection, and the use of counter-screens all facilitate the identification of anti-malarial compounds for lead development or new tools for chemical biology.

**Electronic supplementary material:**

The online version of this article (doi:10.1186/s12936-016-1313-7) contains supplementary material, which is available to authorized users.

## Background

Plants provide prototype molecules and novel templates for drug development, especially for the treatment of malaria [1]. The World Health Organization continues to approximate the global risk for infection with malaria as more than three billion people. Thus, malaria remains one of the world’s more significant health problems [2, 3]. In a recent review of natural products and their effect on the development of anti-malarial drugs, Fernández-Álvaro et al. warn against discounting the use of natural products in the discovery process, citing several examples from plants as supporting evidence [4]. The current portfolio of anti-malarials contains several combinations based upon artemisinin, a natural product (NP) molecule. Additionally quinine, a natural product, and the aminoquinolines, based upon natural products, remain mainstays of therapy. Recently, St Laurent et al. reported that artemisinin-resistant *Plasmodium falciparum* parasites are rapidly spreading in Southeast Asia [5], necessitating a steady pipeline of novel anti-malarial agents [6]. Consequently, this project focused resources on screening NPs for new leads that target the malaria parasite.

NP drug discovery screening programmes face many challenges, including the presence of low quantities of an active constituent in crude extracts, interfering compounds present within extracts, and influence of multiple active or toxic compounds. These factors may lead to unnecessary purification efforts by encouraging researchers to follow misleading hits. Recently, a new strategy has been developed to avoid these challenges: prefractionation of plant extracts coupled with high throughput screening. In 2008, Wagennar et al. described the establishment of a pre-fractionated natural products library at Wyeth using reversed-phase HPLC to complement their existing library of crude extracts [7]. In 2014, Avery et al. identified potential leads against blood-stage *P. falciparum* via screening and hit evaluation of a medium-sized chemical library [8].

During this period, an adaptation of the NPs drug discovery fractionation model was reported [9] using automated high-throughput systems (AHTS), in which the crude extracts of plants are fractionated by using various chromatography methods prior to primary screening [10, 11]. Pre-fractionation enriches secondary metabolites by removing salts, sugars, lipids, tannins, and other molecules, thereby facilitating the detection of minor active compounds. The fractionation procedure is designed to collect fractions that are enriched for milligram amounts of secondary metabolites with physiochemical properties bridging lead-like and drug-like.

In this platform, analytical (QC) data for column fractions is gathered simultaneously with fractionation using ultra-performance liquid chromatography (UPLC) coupled with multiple detectors including an evaporative light scanning detector (ELSD), UV/VIS photodiode array (PDA), and electrospray ionization mass spectrometer (ESI–MS).

The collection of analytical data facilitates the dereplication of known compounds and allows researchers to prioritize costly downstream purification efforts, focusing on novel bioactive natural products. As crude plant extracts continue to be fractionated during this process, the diversity of represented plant species expands as well. This growth gives us the opportunity to track other effects that the model might have on issues outside drug discovery but relevant to all natural products discovery. The environmental pressures (e.g., deforestation, urbanization, recollection practices) that threaten the existence of pharmacologically important plant species has been recently reviewed [12]. In this regard, the collaboration enables us to underline the importance of evaluating this category of endangered or threatened plant species for this current and future studies so that these resources might be conserved worldwide.

This report addresses using the AHTS method as a platform with UPLC-ELSD-PDA-MS analysis to identify potential leads against *P. falciparum*. In vitro screening of the resulting natural product library of column fractions generated secondary hits from a diverse set of plant extracts. To demonstrate the platform’s utility for anti-malarial drug discovery, the active components from the fractions of two plants, *Berberis thunbergii* and *Eugenia rigida*, were identified by compound dereplication of their UPLC-ELSD-PDA-ESI–MS results and direct isolation of the compounds from scaled extracts.

## Methods

### Plant materials

The aerial parts of *Berberis thunbergii* (Berberidaceae) were collected in Wisconsin by Mr. Andrew Townesmith on August 17, 2005, and a collection specimen (#268) was deposited at the Herbarium of Missouri Botanical Gardens in St. Louis, MO; the leaves of *Eugenia rigida* were collected from Guanica, Puerto Rico in March, 2006. The sample was identified by Mr. F. Axelrod, and a voucher specimen (#3008783; collection #Gust 1116) was deposited at the Herbarium of Missouri Botanical Gardens.

### Extraction and UPLC-ELSD-PDA-MS analysis

Detailed procedures for using AHTS to generate the natural product library have been described [10, 11]. The UPLC-ELSD-PDA-MS data were obtained from a Waters Acquity UPLC-MS system (Waters Corp., Milford, MA). An Acquity UPLC BEH C_18_ column (2.1 × 50 mm, 1.7 μm) was used. The mobile phase gradient used water containing 0.1 % formic acid and acetonitrile. The total run time for each analysis was 3.0 or 5.0 min (Table 3). Ionization and detection of natural products were carried out on a Waters SQ mass spectrometer using both the positive and negative ESI modes. The capillary voltage was set at 3.4 kV. The extractor voltage was 2 V. Nitrogen was used as the nebulizing gas, and the source temperature was set at 130 °C. The scanning was optimized for a *m*/*z* range of 130–1400 Da. A comparison of the profiles indicated minor shifts in peak retention times (t_R_) because the detection was performed on a hyphenated four-channel system (i.e., ELSD or MS profiles ∆t_R_ = 0.03–0.04 min beyond PDA peak maxima). Because of possible differences in ionization efficiencies, the ELSD chromatogram is more accurate in assessing relative contents of individual compounds in any mixture than ESI–MS. Data processing was automatically performed with OpenLynx by extracting all graphic information, such as retention time and UV and ELSD peak areas, and converted to text to allow its transfer to a database for storage and analysis. Each 384-well plate could be analysed in 20 h.

### Antiparasitic assay

The two *P. falciparum* strains used in this study were provided by the MR4 unit of the American type culture collection (ATCC, Manassas, VA, USA): chloroquine-sensitive strain 3D7 and chloroquine-resistant strain K1. Asynchronous parasites were maintained in culture by using the method of Trager and Jensen [13]. Parasites were grown in the presence of fresh, group O-positive erythrocytes (Key Biologics, LLC, Memphis, TN) in Petri dishes at a haematocrit of 4 % in RPMI-based media (RPMI 1640 supplemented with 0.5 % AlbuMAX II, 25 mM HEPES, 25 mM NaHCO_3_ [pH 7.3], 100 µg/mL hypoxanthine, and 5 µg/mL gentamycin). Cultures were incubated at 37 °C in a gas mixture of 90 % N_2_, 5 % O_2_, 5 % CO_2_. For EC_50_ determinations, 20 µL of RPMI 1640 containing 5 µg/mL gentamycin were dispensed in each well of an assay plate (Corning 384-well microtiter plate, clear bottom, tissue culture–treated, catalog no. 8807BC). DMSO compound stocks (60 nL) that had been serially diluted in a separate 384-well white polypropylene plate (Corning, catalog no. 8748BC) were dispensed to the assay plate by hydrodynamic pin transfer (FP1S50H, V&P Scientific Pin Head). Then, 20 µL of a synchronized culture suspension (1 % rings, 4 % haematocrit) was added per well, making the final haematocrit and amount of parasitaemia 2 and 1 %, respectively. Assay plates were incubated for 72 h, and the parasitaemia was determined by a method previously described [14]. Briefly, 10 µL of the following solution in PBS (10 × Sybr Green I, 0.5 % v/v triton, 0.5 mg/mL saponin) was added per well. Assay plates were shaken for 1 min, incubated in the dark for 90 min, and then absorbance was read with the Envision spectrophotometer at 485-nm excitation and 535-nm emission detection. High-throughput assay data were analysed by using the Robust Interpretation of Screening Experiments (RISE) application written in Pipeline Pilot (Accelrys, v. 8.5) and the R program [15]. EC_50_ dose–response curves were fit as described by Stewart et al. [16].

### Cytotoxicity assay

The human cell lines BJ and HEK293 (purchased from the American Type Culture Collection, ATCC, Manassas, VA, USA) were cultured according to ATCC recommendations. BJ is a stable cell line of normal human foreskin fibroblasts; HEK293 is a human embryonic kidney cell line. Approximately 1000 BJ and 400 HEK293 exponentially growing cells were plated per well (in 30 μL) in white polystyrene, sterile flat-bottom 384-well tissue culture–treated plates (Corning, Tewksbury, MA, USA) and incubated overnight at 37 °C in a humidified 5 % CO_2_ incubator. Compound or fraction stock solutions (in DMSO) were pin-transferred (V&P Scientific, San Diego, CA, USA) the following day. Plates were returned to the incubator for 72 h and equilibrated at room temperature for 20 min before addition of 25 μL Cell Titer Glo (Promega) to each well. Plates were shaken on an orbital shaker for 2 min at 500 rpm. Luminescence was read on an Envision plate reader (Perkin Elmer, Waltham, MA, USA) after 15 min. EC_50_ values were calculated with RISE by using a four-parameter logistic equation.

### Phylogenetic analysis of screening activities

A phylogenetic tree of all screened genera was derived from both formal taxonomic classifications (e.g., family, order, class) and informal clades (e.g., rosids, asterids) was constructed. A plot was generated in the R Statistical Computing Environment [15] by using the ‘ggplot2’ graphics packages [17]. Relationships were deduced directly from the phylogenetic classifications within from the NCBI Taxonomy Database [18].

### General compound isolation procedures

UV spectra in methanol were obtained by using a Hewlett-Packard 8453 UV/VIS spectrometer. IR spectra were obtained by using a Bruker Tensor 27 instrument (Bruker Corporation, Billerica, MA, USA). NMR spectra in deuterated chloroform or methanol were acquired on either a 600 MHz Varian Spectrometer at 600 (^1^H) and 150 MHz (^13^C), a Bruker Avance DRX—500 instrument at 500 (^1^H) and 125 MHz (^13^C), or a Varian Mercury 400 MHz spectrometer at 400 (^1^H) and 100 MHz (^13^C), using the residual non-deuterated solvent as an internal standard. Multiplicity determinations (1D) and 2D NMR spectra were obtained by using standard Bruker pulse programs. The progression of compound purification was monitored by using an Agilent 1290 Infinity UHPLC-MS (Agilent Technologies, Santa Clara, CA, USA) and a methanol gradient in 0.05 % formic acid passed through a Waters Acquity BEH C_18_ column (Waters Corporation, Milford, MA, USA, 150 × 2.1 mm, 1.7-µm particle size). TLC was carried out on pre-coated silica gel 60 F_254_ (EMD Chemicals Inc., Darmstadt, Germany) by using dichloromethane-methanol-ammonia (8:2:0.1) as the solvent system. Centrifugal preparative TLC (CPTLC, using a Chromatotron^®^, Harrison Research Inc., Palo Alto, CA, USA, model 8924) was carried out on 2- or 4-mm silica gel P_254_ (Analtech) rotors. Reverse mode CPTLC was completed on a custom-made 6-mm RP C18 silica gel Chromatorotor™ (i.e., 3 g indicator was added per 100 g C18 powder) [19]. Samples were dried by using a Savant Speed Vac Plus SC210A concentrator. The compounds were visualized by spraying the TLC plates with either Dragendorff’s reagent or vanillin-sulfuric acid.

### Extraction and isolation of alkaloids from *Berberis thunbergii*

The powdered, air-dried aerial parts of *B. thunbergii* (100 g) were extracted by percolation with 95 % ethanol (250 mL × 3, 48 h), and the combined ethanolic extracts were evaporated under reduced pressure, then dried (5 g). The crude extract (4 g) was dissolved in aqueous 0.1 N hydrochloric acid (pH 4) and partitioned with chloroform (150 mL × 4); the combined chloroform fraction was concentrated under reduced pressure (1.2 g). The defatted aqueous acidic fraction was made basic by titration of ammonium hydroxide (pH 11) and again partitioned with chloroform (150 mL × 4). The combined chloroform fraction was dried over anhydrous sodium sulfate and concentrated under reduced pressure to yield 0.35 g total alkaloids. The chloroform fraction was subjected to CPTLC to afford compounds **9** (20 mg), **10** (15 mg), and **1** (10 mg) and a mixture of two alkaloids (10 mg) identified as **7** (t_R_ 5.63 min, *m*/*z* 609.2 [M + H]^+^) and **8** (t_R_ 6.19 min, *m*/*z* 609.2 [M + H] ^+^) by UHPLC-MS. Analysis of the basic aqueous fraction by UHPLC-MS revealed compounds **1** (t_R_ 5.03 min, *m*/*z* 336.1), **4** (t_R_ 4.82 min, *m*/*z* 338.1), and **5**/**6** as an unresolved mixture (t_R_ 3.22 min, *m*/*z* 342.1 [M + H]^+^). Compounds **1**, **9**, and **10** were identified by ^1^H- and ^13^C NMR spectroscopic analysis, and **4** and **6**–**8** were identified by UHPLC-MS (see Additional file 1). Finally, the identity of **1** was confirmed by direct comparison with an authentic sample of berberine.

### Extraction and isolation of triterpenes from *Eugenia rigida*

The dried powdered leaves (107 g) were extracted by performing sonication for 2 h in hexane, followed by dichloromethane (each 600 mL × 3). The combined hexane and dichloromethane extracts were filtered and concentrated under reduced pressure, yielding 2.5 g and 3.87 g, respectively. A portion of the *n*-hexane extract (2 g) was subjected to CPTLC in reverse mode. The rotor was saturated with 10 % water in methanol, and the sample in acetonitrile was loaded on the disc; left to dry; eluted with a step gradient of water–methanol (from 30 to 0 % water), with fractions being collected by a fraction collector; and then pooled by TLC (silica gel, toluene: ethyl acetate 9:1). Fractions 33–52 and 53–60 were eluted with 15 % water in methanol and found to be active. Therefore, fraction 33–52 (B) was subjected to CPTLC over a silica gel disc and eluted with toluene, yielding a sub-fraction B6-9 (67 mg), which was separated by another 2-mm silica gel CPTLC disc (solvent: hexane–acetone) to afford **11** (19 mg), **12** (3 mg), and—after another CPTLC purification— compound **13** (11 mg). The structures of compounds **11**–**13** were determined by comparing their ^1^H and ^13^C NMR data with those reported in the literature (see Additional file 1) and further confirmed by direct comparison with their respective authenticated samples.

## Results and discussion

High-throughput screening against chloroquine (CQ)-sensitive (3D7) and -resistant (K1) strains of *P. falciparum* was performed to demonstrate the utility of the plant NP library generated by AHTS fractionation. In vitro cytotoxicity assays were performed by using BJ (human foreskin fibroblast) and HEK293 (human embryonic kidney) cell lines, to allow exclusion of hits toxic to mammalian cells. A total of 16,177 fractions from various parts (i.e., roots, leaves, bark) of 519 plant species were screened for *P. falciparum* growth inhibition revealing 900 active fractions from 35 plants (5.5 % hit rate). Active fractions were subjected to secondary dose–response studies showing EC_50_ values < 10.6 µg/mL against 3D7 or K1 and EC_50_ values of <10.1 µg/mL against HEK293 or BJ cells (Table 1).

**Table 1:** Anti-malarial activity (% activity and EC_50_, μg/mL) of AHTS fractions from *Berberis* *thunbergii* (extract code 80679)

Fraction or COMBI	Primary (% activity)			Secondary (EC_50_, μg/mL)			
	μg/mL	MAR3D7^a^	MARK1^b^	HEK293^c^	BJ^d^	MAR3D7^a^	MARK1^b^
f8/c9	23.68	43.4	37	2.15	−0.85		
f9/c8	35.75	95.2	80.2	2.52	6.63	8.15	7.57
f10/c7	23.68	97.4	95	8.13	4.91	0.53	0.63
f11/c6	14.39	96.9	96.5	5.29	0	2.02	3.6
f12/c5	8.37	92.8	93.4	7.42	0.7	0.61	0.81
f13/c5	5.57	92	92.4	3.59	0.45	0.39	0.45
f14/c4	5.11	89.9	96	5.05	−0.48	0.86	2.07
f15/c4	7.43	84.9	97.6	6.94	0.56	1.11	10.61
f16/c3	11.14	84.4	93.7	10.1	1.13	0.21	4.77
f17/c2	6.96	95.7	94.6	1.09	1.48	1.66	1.7
f18/c2	5.11	76.9	39	3.12	−0.94		
f19/c1	4.18	35.6	21.2	1.29	0.64		

^a^Chloroquine-sensitive *P. falciparum* strain

^b^Chloroquine-sensitive *P. falciparum* strain

^c^Human embryonic kidney cells

^d^Human foreskin fibroblast

**Fig. 1:**
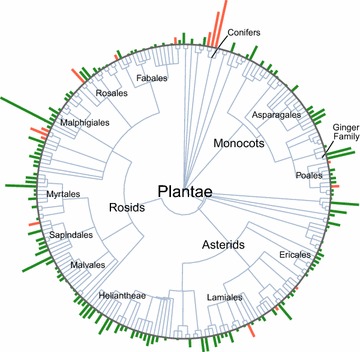
A phylogenetic tree of all screened genera. This tree was derived from both formal taxonomic classifications (e.g., family, order, class) and informal clades (e.g., rosids, asterids). *Bars* on the circumference indicate the total number of hits in each genus against the 3D7 and K1 *P. falciparum* cell lines. Hits were obtained from over 20 different species of the 46 in the entire library that were categorized as endangered or threatened. Genera in which some or all hits were derived from endangered species are marked in *red*. Larger groups, including many of the orders containing ≥7 genera and sub-order nodes that contained highly productive genera, are labelled

The phylogenetic tree in Fig. 1 demonstrates the substantial diversity of plant genera yielding anti-plasmodium activity from the natural product library. Among these actives, species considered endangered or threatened for extinction accounted for 123 of the 900 fraction hits—accounting for more than 13 % of all hits having greater than 50 % inhibition in either *P. falciparum* strain tested—reinforcing the need to conserve these resources globally.

All fractions generated from these plants had EC_50_ value ranges of 0.21–50.28 µg/mL against 3D7 and 0.08–20.04 µg/mL against K1 strains. The AHTS COMBIs (combined fractions) 80679-c3–80679-c8 from *B. thunbergii* and COMBI 79575-c5 obtained from *E. ridiga* had activity against *P. falciparum* (Table 1). To obtain proof-of-concept that the platform faithfully returns known natural products from fraction collections, dereplication was carried out for these samples, with analysis of the LC–MS data being compared to the published literature enabling the prediction of compounds present in these fractions [20].

### UPLC-ELSD-PDA-MS analysis of *B. thunbergii*-active COMBIs

*Berberis thunbergii*, known as Japanese barberry (Berberidaceae), is a deciduous shrub native to eastern Asia, including Japan [21]. It is widely grown as an ornamental plant, both in Japan and elsewhere in the temperate areas of the northern hemisphere. Several chemical investigations of this species have described the presence of protoberberine (i.e., berberine) and isoquinoline (i.e., berbamine) alkaloids, some of which were later shown to possess in vitro anti-malarial activities [22].

**Fig. 2:**
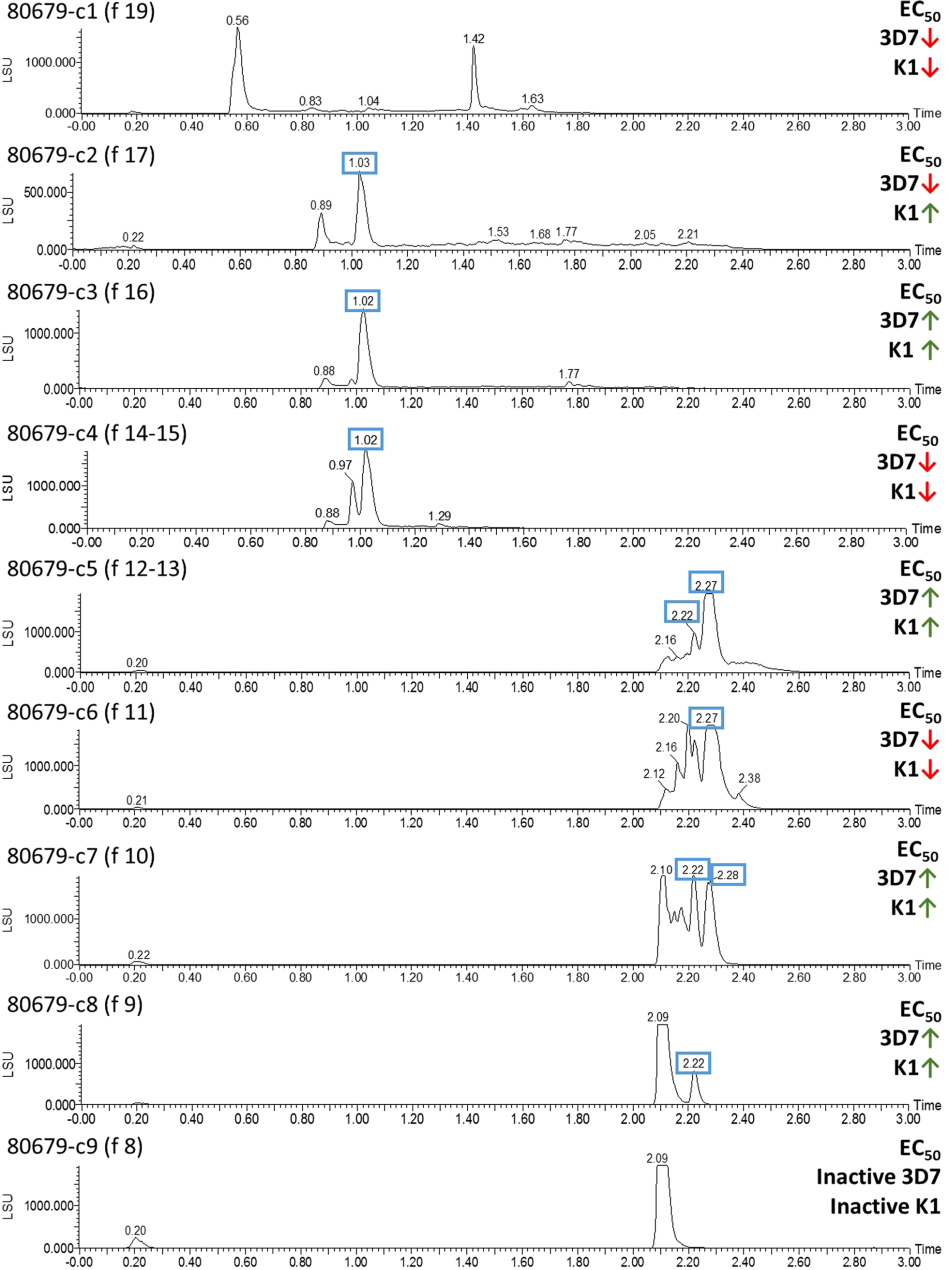
ELSD chromatograms of *Berberis thunbergii* fractions 80679-c1–80679-c9. UPLC conditions (details in Additional file 1): Fraction 80679-c1 injected by using “3 min_nonpolar_NP” method. Fractions 80679-c2–80679-c4 injected by using “3 min_NP” method. Fraction 80679-c5 injected by using “NP” method. Fractions 80679-c6–80679-c9 injected by using “3 min_polar_NP” method. Anti-plasmodium activity of the COMBI is expressed as a *green* or *red arrow* on the basis of its potency compared to its preceding fraction(s) in COMBIs, starting from the most hydrophilic fraction at the bottom (f8) and continuing to the most lipophilic (f19) fraction at the *top* of the figure

A total of seven active COMBIs (80679-c2–80679-c8) derived from AHTS fractionation of the ethanolic extract had inhibitory activity against *P. falciparum* 3D7 and K1 strains, with EC_50_ values of 0.21–8.15 µg/mL and 0.45–10.61 µg/mL, respectively (Table 1). These COMBIs were devoid of cytotoxicity against both BJ and HEK293 cell lines. In Fig. 2, peaks at t_R_ 1.02 min in the ELSD chromatogram were shown in fractions 80679-c2–80679-c4, and peaks at t_R_ 2.22–2.27 min were shown in fractions 80679-c5–80679-c8. In the inactive fractions, 80679-c1 and 80679-c9, these peaks were not detected. Therefore, compounds in these peaks were predicted to be the active constituents of those COMBIs.

**Fig. 3:**
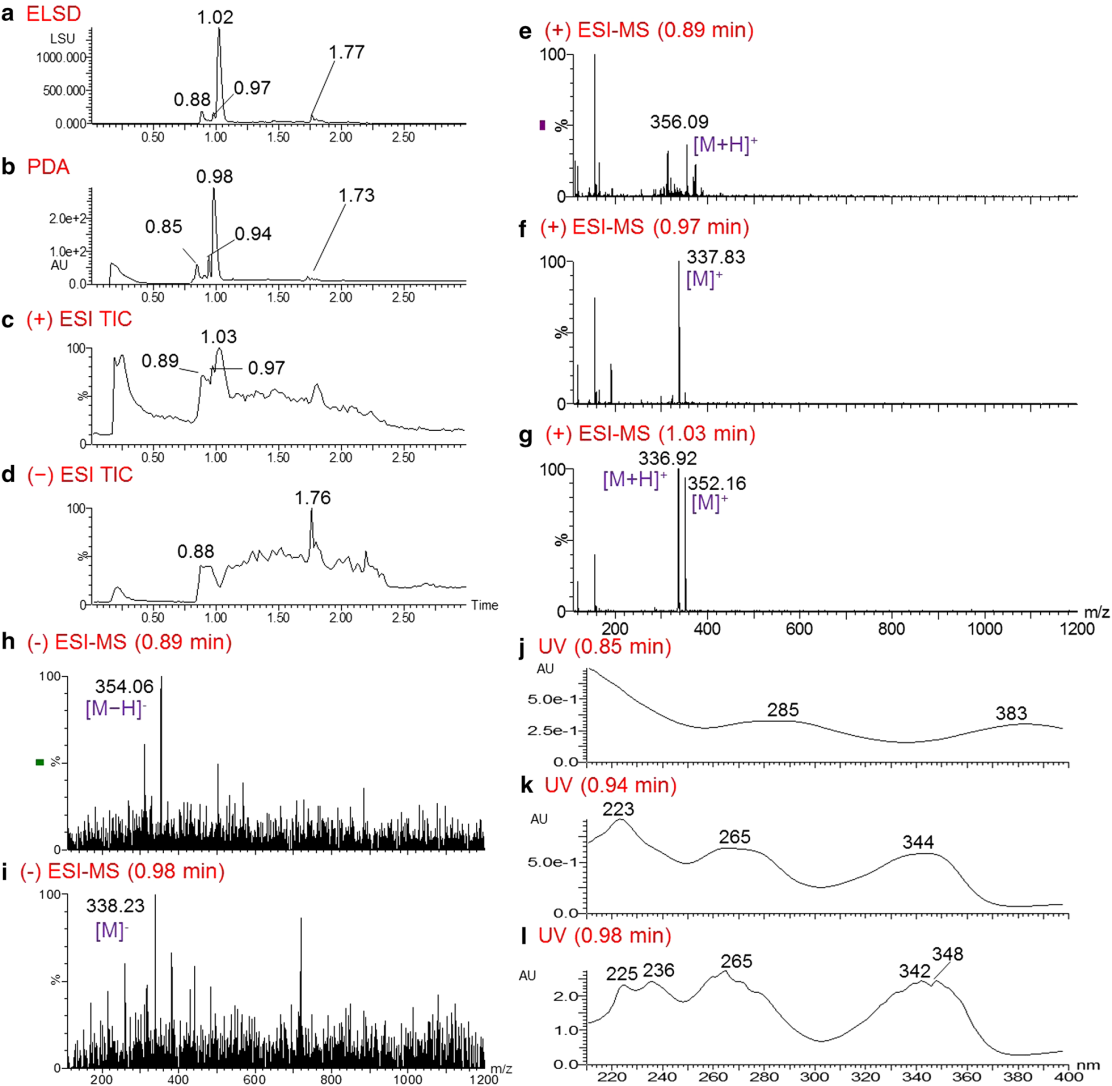
UPLC-MS-ELSD-PDA analysis of fraction 80679-c3 of *Berberis thunbergii*. **a** ELSD chromatogram; **b** PDA chromatogram; **c** positive ESI–MS TIC chromatogram; **d** negative ESI–MS TIC chromatogram; **e**–**g** positive ESI–MS spectra of the compounds with respective retention times of 0.85, 0.94, and 0.98 min in the PDA chromatogram; **h**, **i** negative ESI–MS spectra of the compounds with respective retention times of 0.85 and 0.94 in the PDA chromatogram; **j**–**l** UV spectra of the compounds with respective retention times of 0.85, 0.94, and 0.98 min in the PDA chromatogram. UPLC conditions: acquity UPLC BEH C_18_ column (2.1 × 50 mm, 1.7 μm); gradient elution with 0– 100 % ACN in 0.1 % HCOOH in 3 min

COMBI 80679-c3 had the most potent activity against 3D7, with an EC_50_ value of 0.21 µg/mL. In this COMBI, four main peaks are shown in the PDA chromatogram at t_R_ values of 0.85, 0.94, 0.98, and 1.73 min. Positive ESI–MS results showed good ionization efficiency for most of the fractions of *B. thunbergii*, which are rich in alkaloids. The most intense peak (t_R_ = 0.98 min) in the PDA chromatogram correlated with two quasi-molecular ions at *m*/*z* 336.92 [M]^+^ and *m*/*z* 352.16 [M]^+^ (Fig. 3g) in the positive ESI–MS spectrum, indicating the presence of berberine (**1**) (MW 336) and palmatine (**2**) (MW 352). The comparison of their UV spectra also indicated that the peak at t_R_ 0.98 min was composed of a mixture (Fig. 3l). In addition, the two strong ion spectra peaks at *m*/*z* 356.09 [M + H]^+^ (Fig. 3e) and *m*/*z* 337.83 [M]^+^ (Fig. 3f) were generated by positive ESI–MS from peaks at t_R_ 0.85 and 0.94 min, respectively. However, the corresponding weak peaks at *m*/*z* 354.06 [M − H]^−^ and *m*/*z* 338.22 [M]^−^ were detected in the negative ESI–MS because of the low ionization of these two alkaloids (Fig. 3h, i). The UV spectra displayed absorptions of the compound with MW 355 at 285 and 383 (sh) nm (Fig. 3j) and those of the compound with MW 338 at 223, 265, and 344 (sh) nm (Fig. 3k). On the basis of collective QC data and published values, these two compounds were predicted to be glaucine (**3**) and columbamine (**4**), respectively. Compounds **1**-**3** were isolated previously from *B. thunbergii* [22], and **4** was isolated from multiple species, including *Berberis vulgaris* [23, 24].

**Fig. 4:**
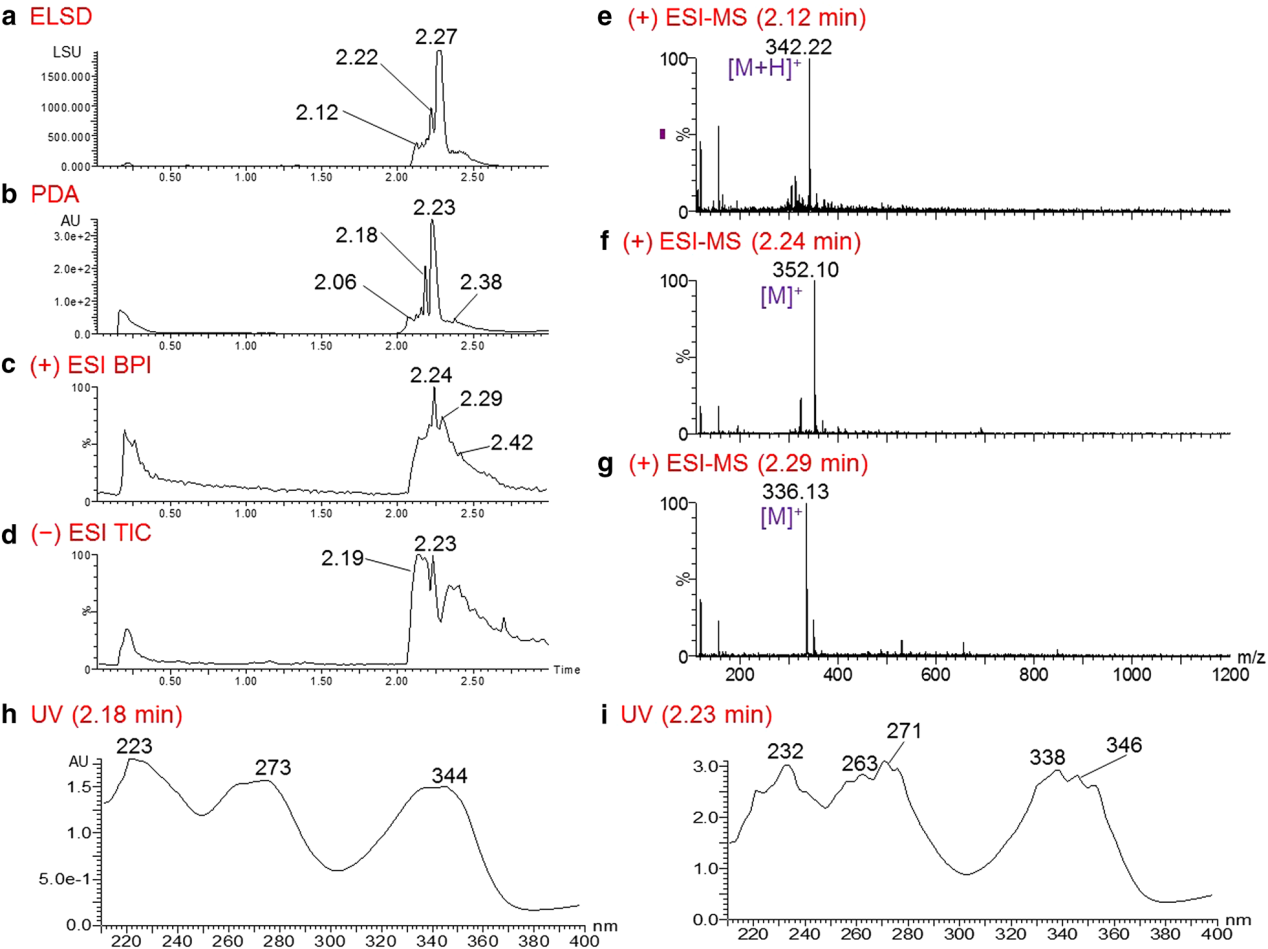
UPLC-MS-ELSD-PDA analysis of fraction 80679-c5 of *Berberis thunbergii*. **a** ELSD chromatogram; **b** PDA chromatogram; **c**, **d** positive ESI–MS BPI and negative ESI–MS TIC chromatograms, respectively; **e**–**g** positive ESI–MS spectra of the compounds with respective retention times of 2.06, 2.18, and 2.23 min in the PDA chromatogram; **h**, **i** UV spectra of the compounds with respective retention times of 2.18 and 2.23 min in the PDA chromatogram. UPLC conditions: acquity UPLC BEH C_18_ column (2.1 × 50 mm, 1.7 μm); gradient elution with 0–100 % ACN in 0.1 % HCOOH in 3 min

COMBI 80679-c5 had strong activity against 3D7 and K1 strains (EC_50_ of 0.39 and 0.45 µg/mL, respectively). Analysis of this COMBI gave six dominant peaks in the PDA chromatogram at 2.06, 2.12, 2.16, 2.18, 2.23, and 2.38 min (Fig. 4b). The peaks at 2.23 and 2.18 min in the PDA were respectively identified as berberine (**1**; *m*/*z* 336.13) and palmatine (**2**; *m*/*z* 352.10) (Fig. 4f, g), with UV spectra similar to those of COMBI 80679-c3 (Fig. 4h, i). In addition, the correlating ESI–MS spectra displayed an ion signal at *m*/*z* 342.22 [M + H]^+^ (Fig. 4e) for the peak at 2.06 min. On the basis of the published literature, two compounds with MW 341 could be predicted, thalicmidine (**5**) and isocorydine (**6**), which were reported previously from *B. thunbergii* [22].

**Fig. 5:**
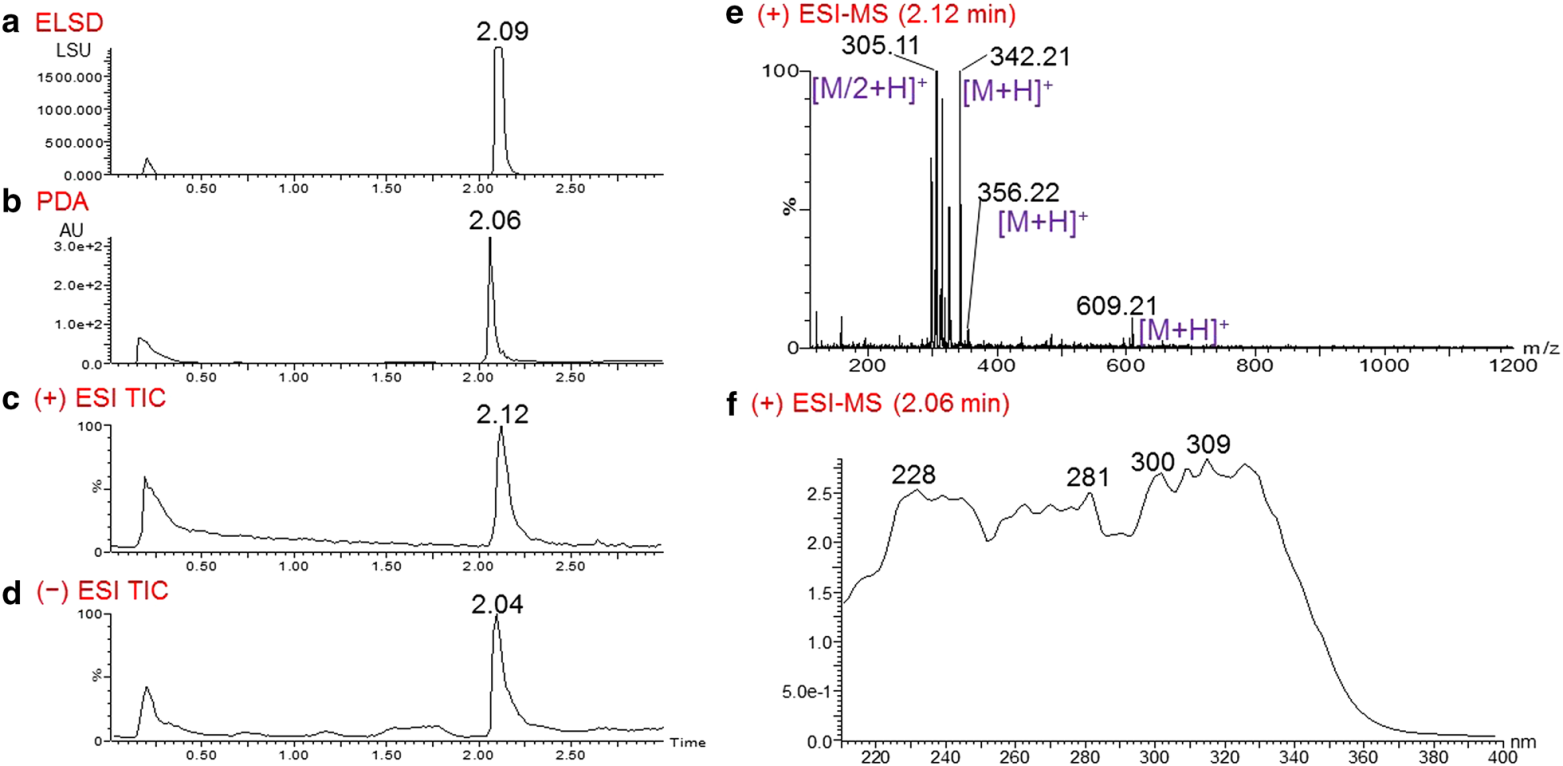
UPLC-MS-ELSD-PDA analysis of fraction 80679-c9 of *Berberis thunbergii*. **a** ELSD chromatogram; **b** PDA chromatogram; **c**, **d** positive and negative ESI–MS TIC chromatograms, respectively; **e** positive ESI–MS spectra of the compound with a retention time of 2.06 min in the PDA chromatogram; **f** UV spectra of the compound with a retention time of 2.06 min in PDA chromatogram. UPLC conditions: Acquity UPLC BEH C_18_ column (2.1 × 50 mm, 1.7 μm); gradient elution with 0–100 % ACN in 0.1 % HCOOH in 3 min

Finally, a single peak at 2.06 min was observed for COMBI 80679-c9 in the PDA chromatogram. A dimeric bis-benzylisoquinoline (BBIQ) alkaloid with MW 608 was predicted according to the quasi-molecular ion shown at *m*/*z* 609.21 [M + H]^+^, together with its monomer fragment at *m*/*z* 305.11 [M/2 + H]^+^ in the corresponding ESI–MS spectrum (Fig. 5e). On the basis of published literature [22, 24, 25], this compound was predicted to be oxyacanthine (**7**) and/or berbamine (**8**) (MW 608). In addition, glaucine (**3**, MW 355) and thalicmidine and/or isocorydine (**5** or **6**, MW 341) were also identified from this COMBI on the bases of the ion spectra peak at *m*/*z* 356.22 [M + H]^+^ and 342.22 [M + H]^+^ (Fig. 5e), respectively.

### UPLC-ELSD-PDA-MS analysis of *Eugenia rigida*-active COMBIs

**Fig. 6:**
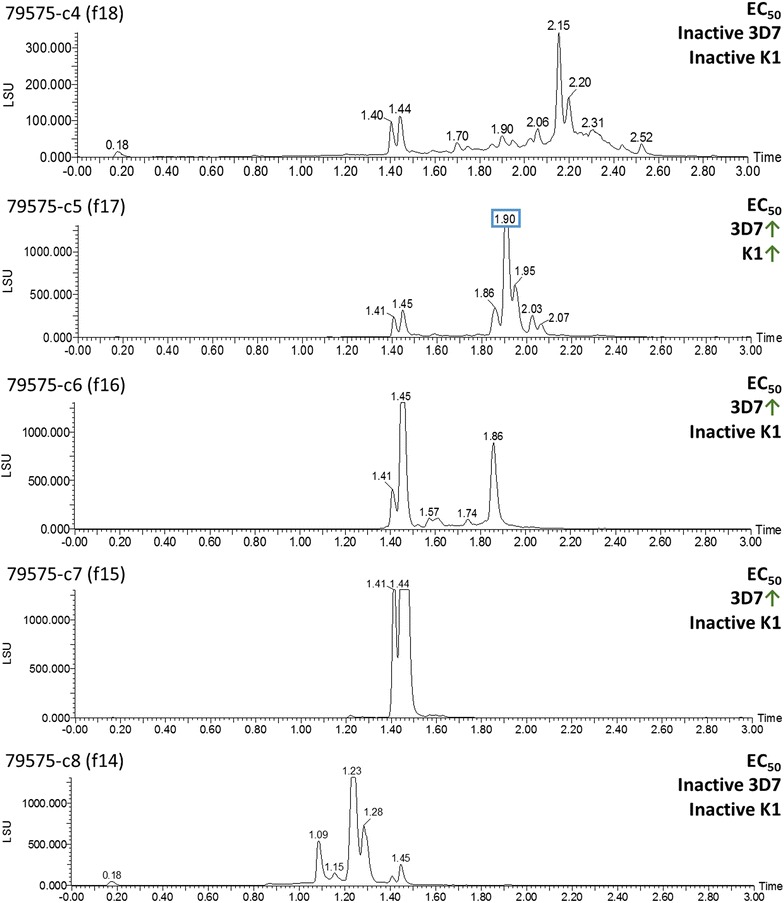
ELSD chromatogram of *Eugenia rigida* fractions 79575-c4–79575-c8. UPLC conditions: fractions all run by using the “3 min_NP” method (details in Additional file 1). Anti-plasmodium activity of the COMBI is expressed as a *green* or *red arrow* on the basis of its potency compared to its preceding fraction(s) in COMBIs, starting from the most hydrophilic fraction at the bottom (f14) and continuing to the most lipophilic (f18) fraction at the *top* of the figure

**Fig. 7:**
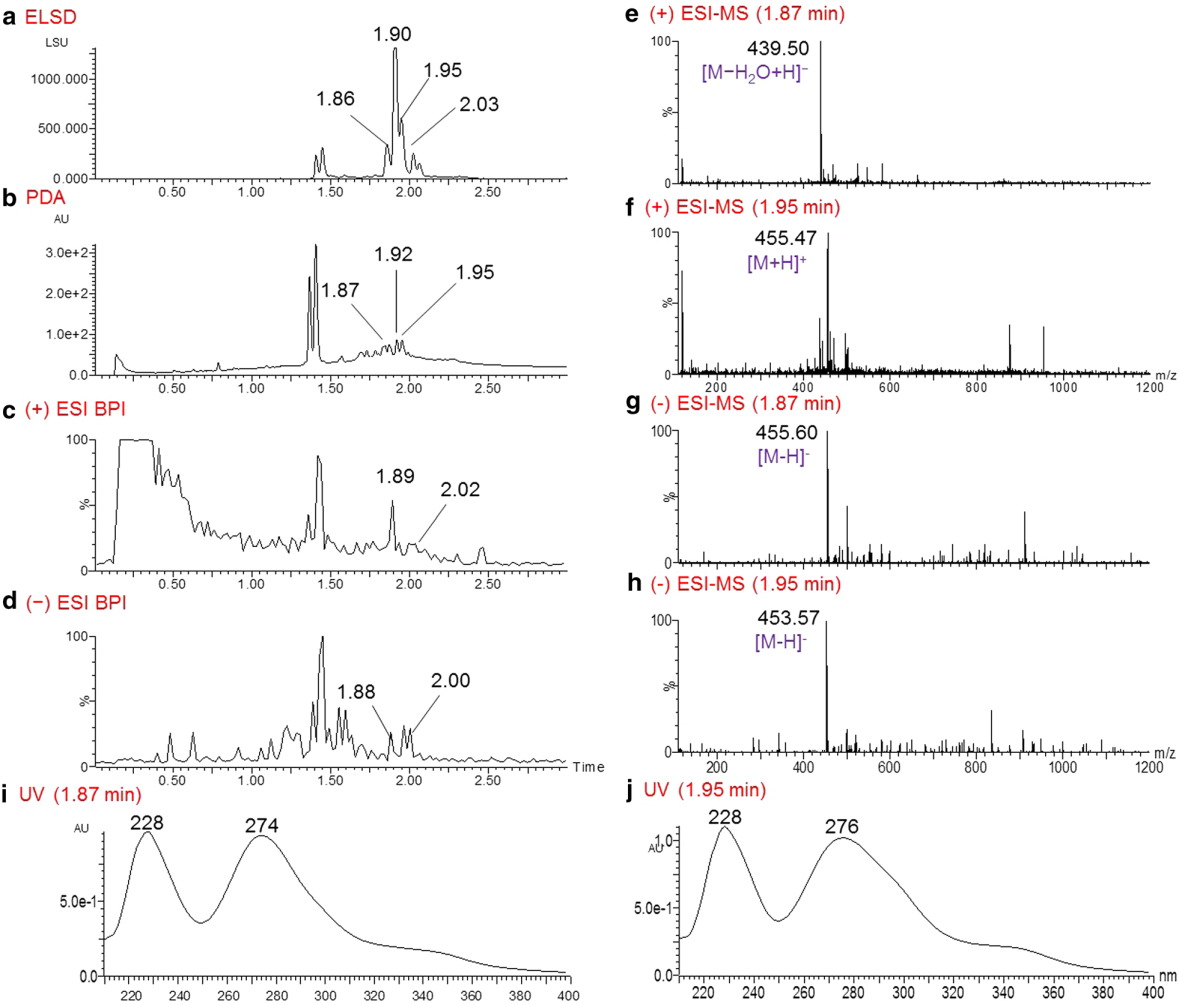
UPLC-MS-ELSD-PDA analysis of fraction 79575-c5 of *Eugenia rigida*. **a** ELSD chromatogram; **b** PDA chromatogram; **c** and **d** positive and negative ESI–MS BPI chromatograms, respectively; **e**, **f** positive ESI–MS spectra of the compounds with respective retention times of 1.87 and 1.95 min in the PDA chromatogram; **g**, **h** negative ESI–MS spectra of the compounds with respective retention times of 1.87 and 1.95 min in the PDA chromatogram; **i**, **j** UV spectra of the compounds with respective retention times of 1.87 and 1.95 min in the PDA chromatogram. UPLC conditions: Acquity UPLC BEH C_18_ column (2.1 × 50 mm, 1.7 μm); gradient elution with 0–100 % ACN in 0.1 % HCOOH in 3 min

*Eugenia rigida* is a shrub or small tree (Myrtaceae) producing characteristic brown and green fruits, which turn black when mature. This plant, present in Brazil and Argentina and distributed in the Caribbean islands, has been used traditionally for the treatment of leukaemias. Earlier investigation of *E. rigida* exhibited (*E*)- and (*Z*)-3,4,3′,5′-tetramethoxystilbenes and 4,3′,5′-trimethoxyresveratrol with cytotoxic and cancer-related signal-modulating activities [26]. The crude ethanolic extract was inactive in the initial screens against *P. falciparum*, but the AHTS-derived COMBI 79575-c5 had potent activity against 3D7 and K1 strains, with respective EC_50_ values of 0.6  and 0.19 µg/mL; other COMBIs were inactive. COMBI 79575-c5 contained a peak at t_R_ 1.90 min in the ELSD chromatogram (Fig. 6), which was not detected in other inactive COMBIs; hence, compounds in this peak were predicted to be the active constituents of COMBI 79575-c5. This COMBI’s QC profile showed seven main peaks in the PDA chromatogram at t_R_ 1.37, 1.41, 1.82, 1.87, 1.92, 1.95, and 2.02 min (Fig. 7b). The positive and negative ESI–MS detectors (Fig. 7c, d) were used for the analysis of compounds that showed good ionization efficiency in these detectors. From the positive- and negative-mode MS spectra at t_R_ 1.87 min, quasi-molecular ions were seen at *m*/*z* 439.50 [M − H_2_O + H]^+^ and 455.60 [M − H]^−^ (Fig. 7e, g), and the corresponding UV spectra displayed absorption bands at 228 and 274 (sh) nm (Fig. 7i). These data indicate the presence of betulinic acid (**12**, MW 456), a pervasive triterpene previously isolated from *Eugenia* species [27]. Accordingly, the compounds detected at t_R_ 1.87 min in 79575-c5 were predicted to be *α*- or *β*-betulinic acid epimers (**11, 13**). Another detected ion with *m*/*z* 455.47 [M + H]^+^ (453.57 [M − H]^−^ for negative MS) (Fig. 7f, h) eluted at t_R_ 1.95 min, indicating the existence of a compound 2 Da smaller than betulinic acid. The associated UV spectra displayed maxima at 228 and 276 (sh) nm (Fig. 7j), similar to betulinic acid, and was thus predicted to be the oxidized derivative betulonic acid (**12**).

### Compound isolation from *Berberis thunbergii* and *Eugenia rigida*

**Fig. 8:**
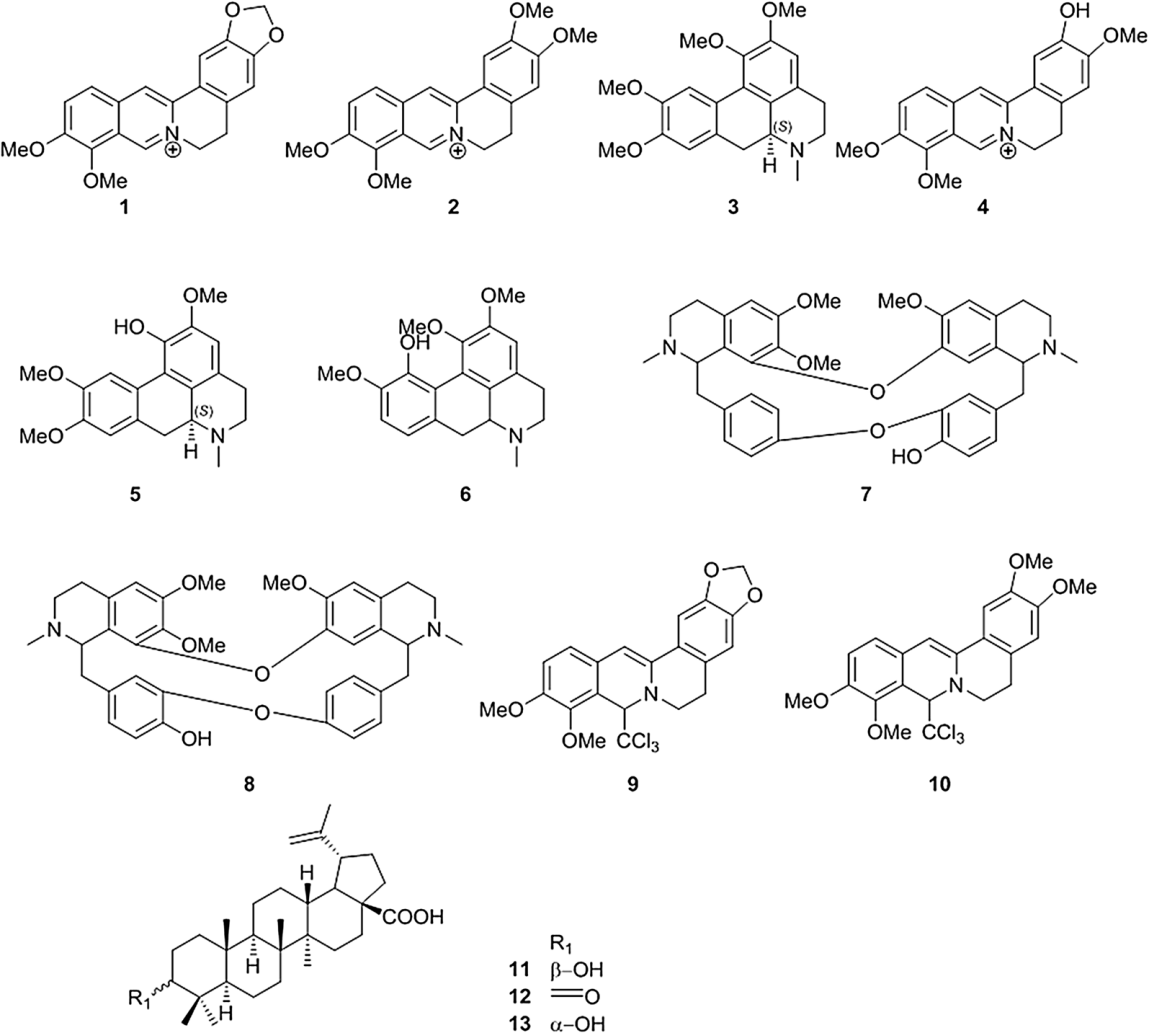
Structure of compounds isolated from *Berberis* *thunbergii* and *Eugenia rigida*

To identify the anti-malarial constituents in *B. thunbergii* and *E. rigida* and validate the discovery platform, scaled isolations were completed from the original plant material to yield several pure NPs (Fig. 8). The results of the TLC analysis of the crude ethanolic extract of *B. thunbergii* were directly compared with seven COMBIs (80679-c2–80679-c8) and with authentic samples of berberine (**1**); the results suggested the presence of compound **1** in the extract, as predicted from UPLC-ELSD-PDA-MS analysis. The extract was subjected to acid–base fractionation to separate the total alkaloid component in the chloroform fraction, which was subjected to CPTLC over a silica gel disc to afford berberine (**1**); a mixture of dimeric BBIQ alkaloids, oxyacanthine (**7**), and berbamine (**8**); 8-trichloromethyldihydroberberine (**9**); and 8-trichloromethyldihydropalmatine (**10**). Compounds **9** and **10** are artifacts formed due to the reactivity of the polar nitrile to nucleophilic attack in the presence of chloroform during acid–base partitioning [28]. An examination of the aqueous fraction obtained from acid–base fractionation showed three major alkaloids, identified by UHPLC/MS as berberine (**1**), columbamine (**4**, MW 338), and thalicmidine (**5**, MW 341) or isocorydine (**6,** MW 341). The structures of **1**, **9**, and **10** were established by evaluating ^1^H- and ^13^C NMR spectroscopic and HRMS data; those of **4** and **6**–**8** were identified by evaluating UHPLC/MS data (Additional file 1).

Compounds **11**–**13** from the active COMBI (79575-c5) of *E. rigidia* were also scaled and isolated. The plant material was successively extracted with *n*-hexane, dichloromethane, and methanol, and the *n*-hexane extract showed the presence of similar compounds upon comparison of its TLC profile to that of the active COMBI. Subsequently, all three compounds— *β*-betulinic acid (**11**), betulonic acid (**12**), and *α*-betulinic acid (**13**)— were isolated from the *n*-hexane extract by performing two rounds of CPTLC and using C18 silica in reverse-phase mode followed by silica gel in normal-phase mode. Then, all isolated compounds were identified by analysing their collective spectroscopic data (Additional file 1).

### *Anti*-*malarial* compounds isolated from *Berberis thunbergii* and *Eugenia rigida*

The isolated compounds (**1**, **11**–**13**), mixture of compounds (**7** + **8**), and fractions obtained from *B. thunbergii* and *E. rigida* were tested for in vitro anti-malarial activities against *P. falciparum* (Table 2). Among the fractions from *B. thunbergii*, the most prominent activity was displayed by the basic chloroform partitions. The protoberberine alkaloid berberine (**1**) exhibited strong activity against 3D7 and K1 strains of *P. falciparum*, with respective EC_50_ values of 0.46 and 0.44 µM, and the mixture of dimeric BBIQ alkaloids (**7** + **8**) exhibited strong activity, with an EC_50_ value of 0.42 µM against the K1 strain. Both berberine (**1**) and berbamine (**8**) have reported anti-malarial-associated activities [23, 29], and they appear to be the main active constituents of the COMBI 80679-c5. Although 8-trichloromethyldihydroberberine (**9**) was isolated as an artifact and was not attributed to a COMBI, it was more active than the parent compound, indicating a potentially key pharmacophore in the class. Finally, the triterpenes **11** and **13** that were isolated from *E. rigida* had similar activity against the 3D7 (EC_50_ = 7.7 and 0.3 µM, respectively) and K1 (EC_50_ = 4.0 and 1.1 µM, respectively) strains of *P. falciparum*. The oxidized derivative, betulonic acid (**12)**, had little to no effect on the parasite. Betulinic acid and its derivatives have been reported to have anti-malarial activity in vitro [30] and have reduced parasitemia in mice [31]. Together, compounds **11** and **13** were predicted from the analysis of UPLC-ELSD-PDA-MS profiles of the active COMBI 79575-c5, validating the efficiency of the AHTS fractionation platform for the discovery of anti-malarial NPs.

**Table 2:** Anti-malarial activity of compounds **1** and **9–13** and fractions from *Berberis thunbergii* and *Eugenia rigida* against *P. falciparum*

Sample	Unit	*P. falciparum* EC_50_		Sample	*P. falciparum* EC_50_	
		3D7	K1		3D7	K1
BT-EtOH^a^	μg/mL	NT^g^	NT	ER-EtOH^h^	>100	>100
BT-P-A^b^	μg/mL	0.25	0.291	ER-MeOH^i^	2	1.37
BT-P-B^c^	μg/mL	0.23	0.19	ER-Hex^j^	>100	>100
BT-P–C^d^	μg/mL	NT	NT	ER-Hex-33-52^k^	1	0.6
BT-P-D^e^	μg/mL	10	10	ER-Hex-53-60^l^	0.7	0.6
**7** **+** **8** ^f^	μg/mL	100	0.42	ER-EtOAc^m^	>100	>100
**1**	μM	0.46	0.44	**11**	7.7	4.0
**9**	μM	0.035	0.15	**12**	NT	NT
**10**	μM	NT	NT	**13**	0.3	1.1

^a^ *BT-EtOH* EtOH extract of *B. thunbergii*

^b^ *BT-P-A* acidic CHCl_3_ fraction of BT

^c^ *BT-P-B* basic CHCl_3_ fraction of BT

^d^ *BT-P-C* EtOAc fraction of BT

^e^ *BT-P-D* aqueous fraction of BT

^f^Mixture of **7** and **8**

^g^ *NT* not tested

^h^ *ER-EtOH* EtOH extract of *E. rigida*

^i^ *ER-MeOH* MeOH extract of ER

^j^ *ER-Hex* *n*-hexane extract of ER

^k^ *ER-Hex-33-52* RP-CPTLC fraction of ER

^l^ *ER-Hex-53-60* RP-CPTLC fraction ER

^m^ *ER-EtOAc* EtOAc extract of *E. rigida*

## Conclusions

The use of AHTS enabled the rapid identification of anti-malarial active compounds from plant extracts by analysing QC data generated from UPLC-ELSD-PDA-MS of the active fractions. Moreover, validation of the anti-malarial discovery platform was completed through bioassay-guided isolation of the main active constituents and confirmation of their activities. The resolving power of UPLC successfully separated the tertiary and quaternary alkaloids as well as the dimeric BBIQs from quaternary protoberberines, as shown from the crude extract of *B. thunbergii*. Thus, the AHTS analytical system produced reliable QC data for alkaloid identification from a complex mixture and enabled library dereplication for either compound procurement or prioritization of purification efforts. Five known alkaloids were isolated (**1**, **7–10**) and identified from the ethanolic extract of *B. thunbergii*, two of which were artifacts from the purification. Palmatine (**2**), glaucine (**3**), and thalicmidine (**5**) were previously reported from *B. thundergii* and dereplicated from the QC data, but activity against *P. falciparum* was tracked to berberine (**1**) and berbamine (**8**), both of which are reported to have anti-malarial activity. Finally, three triterpenes (**11**–**13**) were isolated from *E. rigida,* of which **11** and **13** were identified as active compounds, originally dereplicated from the QC data of one fraction (79575-c5). This diversity-oriented NP collaborative platform has successfully merged traditional pharmacognosy practices with high-throughput technologies, providing a rich dataset from which hits can be identified and advanced. The discovery of new NP anti-malarial leads by using this platform is accordingly limited by the contents of the fraction library itself. As the diversity of represented species expands in the library, so will the coverage of the chemical space provided by Nature.

## Additional file

10.1186/s12936-016-1313-7 Specified UPLC conditions, resulting chromatograms and analytical data including NMR, UV and MS spectra for the purified natural products.

## Abbreviations

AHTS: automated high throughput system
BBIQ: bis-benzylisoquinoline
COMBI: combined fraction represented as “[extract code]-c[relative fraction position]”
ESI: electrospray ionization source
ELSD: evaporative light scattering detector
RISE: robust interpretation of screening experiments
sh: spectra peak shoulder
t_R_: chromatographic peak retention time
UPLC: ultra performance liquid chromatography
