# Supplementary material for: Diversity-oriented natural product platform identifies plant constituents targeting Plasmodium falciparum
Source: Malar J. 2016 May 10;15:270. doi: 10.1186/s12936-016-1313-7 (PMC4863362; doi:10.1186/s12936-016-1313-7)
Supplement: Supplementary file 1 — 10.1186/s12936-016-1313-7 Specified UPLC conditions, resulting chromatograms and analytical data including NMR, UV and MS spectra for the purified natural products. [file 12936_2016_1313_MOESM1_ESM.docx]

**Supporting Information**

Diversity-oriented natural product platform identifies plant constituents targeting *Plasmodium falciparum*

Jin Zhang^1^, John J. Bowling^2^, David Smithson^2,‡^, Julie Clark^2^, Melissa R. Jacob^1^, Shabana I. Khan^1^, Babu L. Tekwani^1,3^, Michele Connelly^2^, Vladimir Samoylenko^1, ‡^, Mohamed A. Ibrahim^1^, Mohamed A. Zaki^1, ‡^, Mei Wang^1^, John P. Hester^1,†^, Ying Tu^2^, Cynthia Jeffries^2^, Nathaniel Twarog^2^, Anang A. Shelat^2^, Larry A. Walker^1,3^, Ilias Muhammad^1^* and R. Kiplin Guy^2^*

1 National Center for Natural Products Research, Research Institute of Pharmaceutical Sciences, School of Pharmacy, The University of Mississippi, University, Mississippi 38677, United States.

2 Department of Chemical Biology and Therapeutics, St. Jude Children’s Research Hospital, Memphis, Tennessee 38105, United States.

3 Department of Biomolecular Sciences and Research Institute of Pharmaceutical Sciences, School of Pharmacy, The University of Mississippi, University, Mississippi 38677, United States.

* Corresponding authors

† Deceased on December 11, 2010.

Corresponding authors: Ilias Muhammad, milias@olemiss.edu; R. Kiplan Guy, kip.guy@stjude.org

|  | Content |
| --- | --- |
| Table S1 | UPLC conditions for *B. thunbergii* and *E. rigida* fractions |
| Fig. 1S | ^1^H NMR spectrum (DMSO, 500 MHz) of compound **1** |
| Fig. 2S | ^13^C NMR spectrum (DMSO, 500MHz) of compound **1** |
| Fig. 3S | ^1^H NMR spectrum (CDCl_3_, 500MHz) of compound **9** |
| Fig. 4S | ^13^C NMR spectrum (CDCl_3_, 500MHz) of compound **9** |
| Fig. 5S | Dept-135 NMR spectrum (CDCl_3_, 500MHz) of compound **9** |
| Fig. 6S | HMQC NMR spectrum (CDCl_3_, 400MHz) of compound **9** |
| Fig. 7S | ^1^H NMR spectrum (CDCl_3_, 500MHz) of compound **10** |
| Fig. 8S | ^1^H NMR spectrum (DMSO, 500 MHz) of compound **11** |
| Fig. 9S | ^13^C NMR spectrum (DMSO, 500MHz) of compound **11** |
| Fig. 10S | Dept-135 NMR spectrum (DMSO, 500MHz) of compound **11** |
| Fig. 11S | HMQC NMR spectrum (DMSO, 400MHz) of compound **11** |
| Fig. 12S | HMBC NMR spectrum (DMSO, 400MHz) of compound **11** |
| Fig. 13S | ^1^H NMR spectrum (Pyridine-*d^5^*, 500MHz) of compound **12** |
| Fig. 14S | ^13^C NMR spectrum (Pyridine-*d^5^*, 500MHz) of compound **12** |
| Fig. 15S | ^1^H NMR spectrum (DMSO, 500 MHz) of compound **13** |
| Fig. 16S | ^13^C NMR spectrum (DMSO, 500MHz) of compound **13** |
| Fig. 17S | Dept-135 NMR spectrum (DMSO, 500MHz) of compound **13** |
| Fig. 18S | ^1^H-^1^H COSY NMR spectrum (DMSO, 400MHz) of compound **13** |
| Fig. 19S | HMQC NMR spectrum (DMSO, 400MHz) of compound **13** |
| Fig. 20S | HMBC NMR spectrum (DMSO, 400MHz) of compound **13** |
| Fig. 21S | UV chromatogram of compounds **7** and **8** mixture at 280 nm |
| Fig. 22S | Positive mode ESIMS TIC of compounds **7** and **8** mixture |
| Fig. 23S | MS spectra of compound **7** at 5.63 min |
| Fig. 24S | MS spectra of compound **8** at 6.19 min |
| Fig. 25S | UV chromatogram of basic aqueous fraction of *Berberis Thunbergii* at 325 nm |
| Fig. 26S | Positive ESIMS TIC of basic aqueous fraction of *Berberis Thunbergii* |
| Fig. 27S | MS spectra of compound **5/6** at 3.22 min |
| Fig. 28S | MS spectra of compound **4** at 4.82 min |
| Fig. 29S | MS spectra of compound **1** at 5.03 min |

**Table S1. UPLC conditions for *B. thunbergii* and *E. rigida* fractions**

| UPLC Method | Time | % Phase A | % Phase B |
| --- | --- | --- | --- |
| **3 min_nonpolar_NP** | 1 | 90 | 10 |
|  | 1.3 | 0 | 100 |
|  | 2.95 | 0 | 100 |
|  | 3.0 | 85 | 15 |
| **3 min_NP** | 1 | 90 | 10 |
|  | 2.05 | 5 | 95 |
|  | 2.95 | 5 | 95 |
|  | 3.0 | 90 | 10 |
| **NP** | 1 | 98 | 2 |
|  | 2.0 | 60 | 40 |
|  | 4.9 | 5 | 95 |
|  | 4.95 | 90 | 10 |
| **3 min_polar_NP** | 1 | 98 | 2 |
|  | 2.0 | 60 | 40 |
|  | 2.95 | 5 | 95 |
|  | 3.0 | 90 | 10 |

Phase A: Water/ 0.1% formic acid

Phase B: Acetonitrile/ 0.1% formic acid

Gradients: All run at 1.0 mL/min.

**Fig. 1S:** ^1^H NMR spectrum (DMSO, 500 MHz) of compound **1**

**Fig. 2S:** ^13^C NMR spectrum (DMSO, 500MHz) of compound **1**


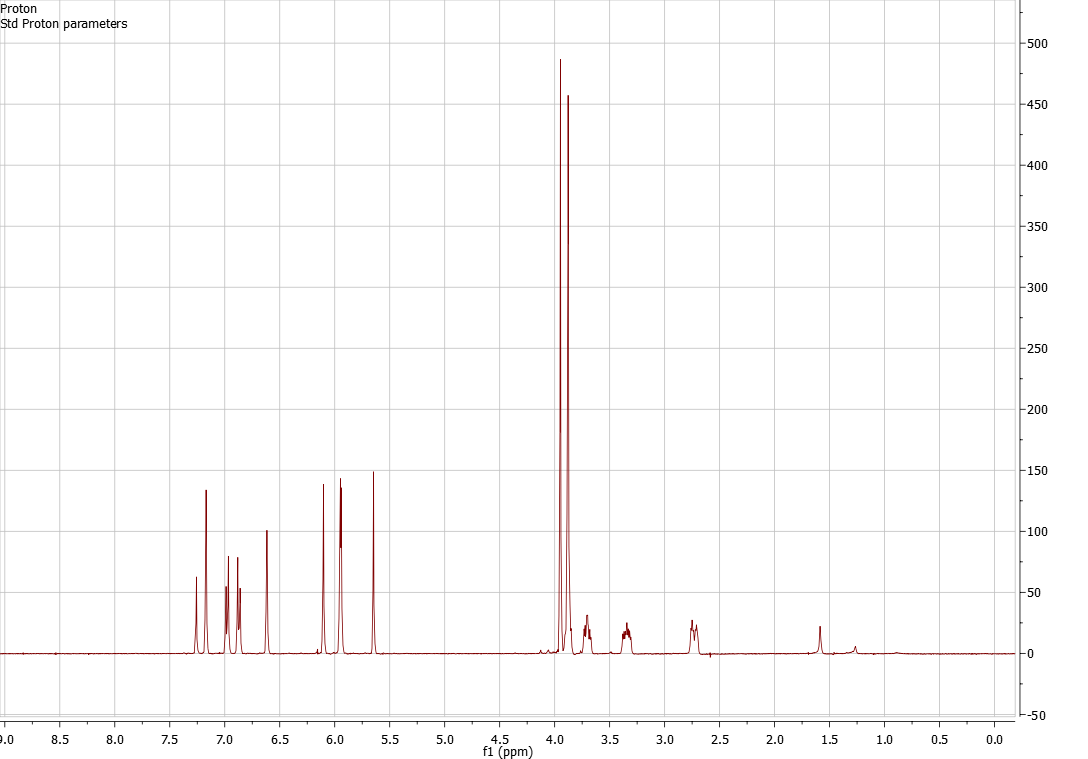


**Fig. 3S:** ^1^H NMR spectrum (CDCl_3_, 500 MHz) of compound **9**


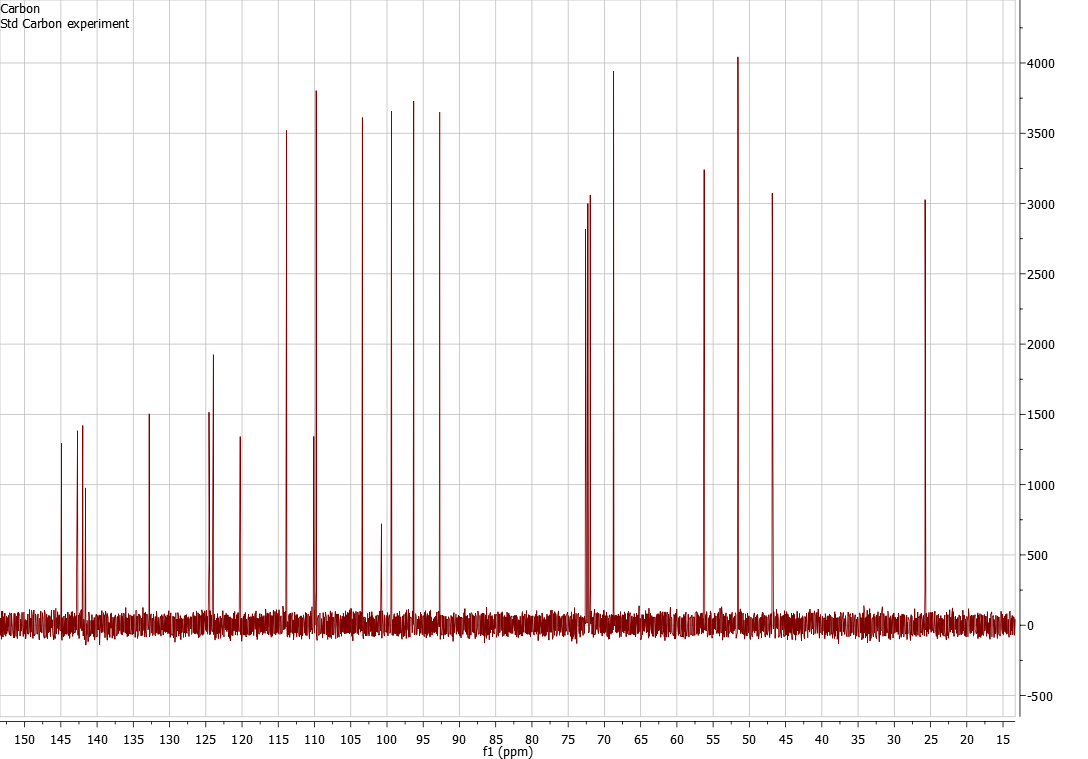


**Fig. 4S:** ^13^C NMR spectrum (CDCl_3_, 500 MHz) of compound **9**


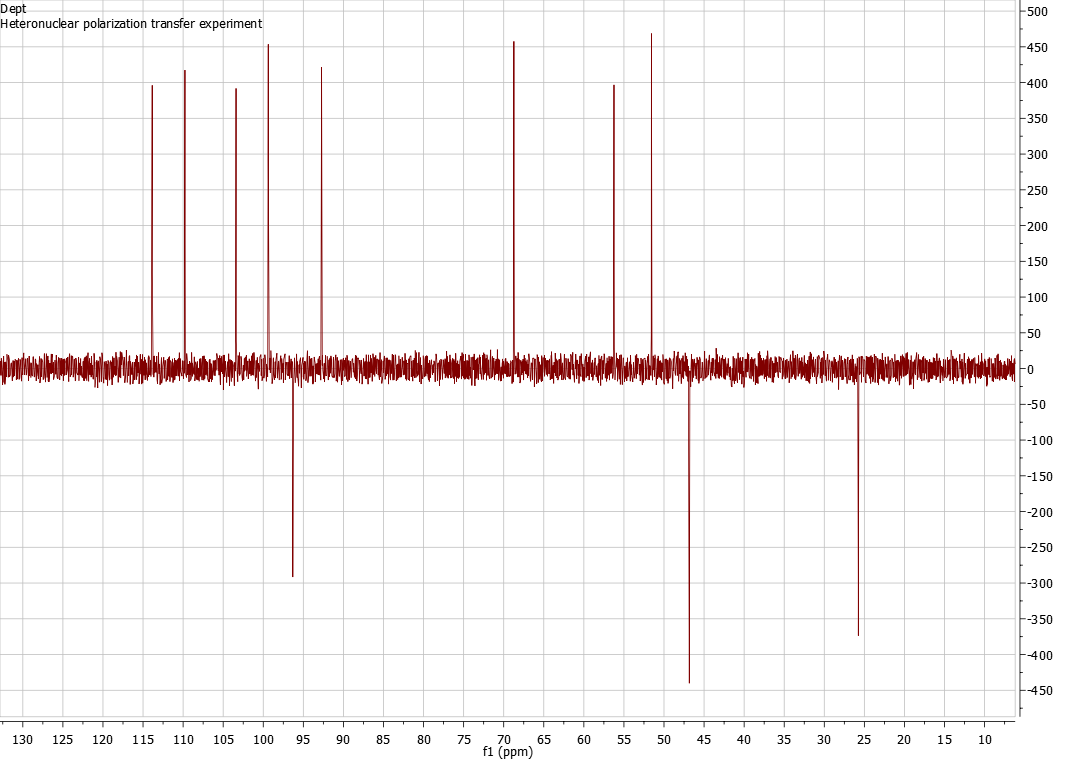


**Fig. 5S:** Dept-135 NMR spectrum (CDCl_3_, 500 MHz) of compound **9**

**
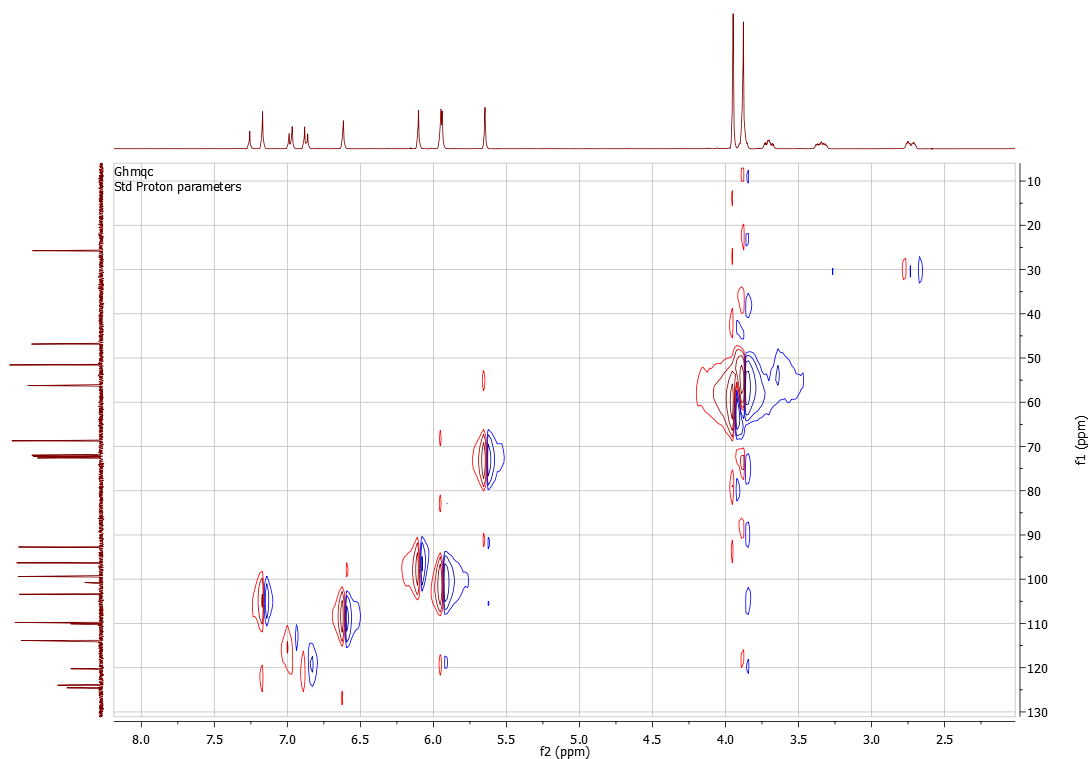
**

**Fig. 6S:** HMQC NMR spectrum (CDCl_3_, 400 MHz) of compound **9**


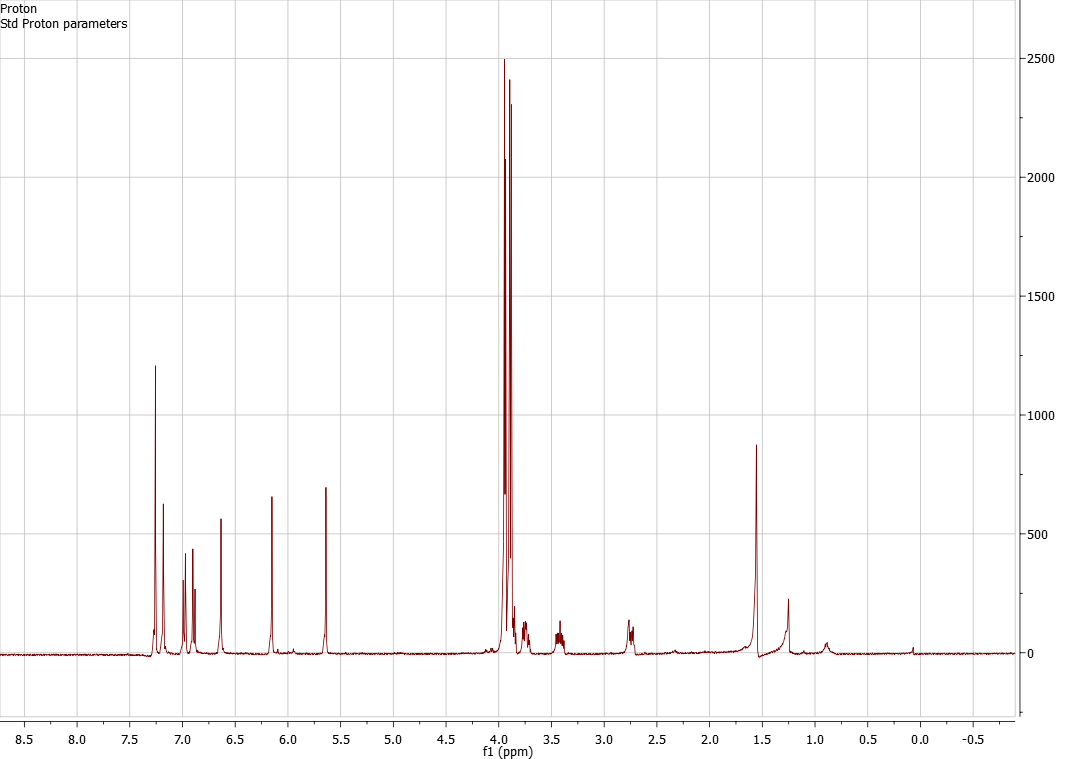


**Fig. 7S:** ^1^H NMR spectrum (CDCl_3_, 500 MHz) of compound **10**

**Fig. 8S:** ^1^H NMR spectrum (DMSO, 500 MHz) of compound **11**

**Fig. 9S:** ^13^C NMR spectrum (DMSO, 500 MHz) of compound **11**

**Fig. 10S:** Dept-135 NMR spectrum (DMSO, 500 MHz) of compound **11**

**Fig. 11S:** HMQC NMR spectrum (DMSO, 400 MHz) of compound **11**

**Fig. 12S:** HMBC NMR spectrum (DMSO, 400 MHz) of compound **11**

**Fig. 13S:** ^1^H NMR spectrum (Pyridine-*d^5^*, 500 MHz) of compound **12**

**Fig. 14S:** ^13^C NMR spectrum (Pyridine-*d^5^*, 500 MHz) of compound **12**

**Fig. 15S:** ^1^H NMR spectrum (DMSO, 500 MHz) of compound **13**

**Fig. 16S:** ^13^C NMR spectrum (DMSO, 500 MHz) of compound **13**

**Fig. 17S:** Dept-135 NMR spectrum (DMSO, 500 MHz) of compound **13**

**Fig. 18S: ^1^H-^1^H** COSY NMR spectrum (DMSO, 400 MHz) of compound **13**

**Fig. 19S:** HMQC NMR spectrum (DMSO, 400 MHz) of compound **13**

**Fig. 20S:** HMBC NMR spectrum (DMSO, 400 MHz) of compound **13**

**Fig. 21S:** UV spectra of compounds **7** and **8** mixture at 280 nm

**Fig. 22S:** Positive ESIMS TIC of compounds **7** and **8** mixture

**Fig. 23S:** MS spectra of compound **7** at 5.63 min

**Fig. 24S:** MS spectra of compound **8** at 6.19 min

**Fig. 25S:** UV spectra of basic aqueous fraction of *Berberis Thunbergii* at 325 nm

**Fig. 26S:** Positive ESIMS TIC of basic aqueous fraction of *Berberis Thunbergii*

**Fig. 27S:** MS spectra of compound **5/6** at 3.22 min

**Fig. 28S:** MS spectra of compound **4** at 4.82 min

**Fig. 29S:** MS spectra of compound **1** at 5.03 min
